# Developing a predictive model for clinically significant prostate cancer by combining age, PSA density, and mpMRI

**DOI:** 10.1186/s12957-023-02959-1

**Published:** 2023-03-07

**Authors:** Zengni Ma, Xinchao Wang, Wanchun Zhang, Kaisheng Gao, Le Wang, Lixia Qian, Jingjun Mu, Zhongyi Zheng, Xiaoming Cao

**Affiliations:** 1 Department of Urology, The Fifth People’s Hospital of Datong, 037000 Datong, China; 2 School of Public Health , Shanxi Medical University, Taiyuan, 030000 China; 3 Department of Nuclear Medicine, Shanxi Bethune Hospital, Taiyuan, 030000 China; 4 Department of Urology, First Hospital of Shanxi Medical University, Taiyuan, 030000 China; 5 Department of Radiology, First Hospital of Shanxi Medical University, Taiyuan, 030000 China; 6 Department of Radiology, Shanxi Bethune Hospital, Taiyuan, 030000 China; 7 Department of Urology, Shanxi Cancer Hospital, Taiyuan, 030000 China

**Keywords:** Clinically significant prostate cancer, mpMRI, PI-RADS v2.1, PSA density, Prostate biopsy, Predictive model

## Abstract

**Purpose:**

The study aimed to construct a predictive model for clinically significant prostate cancer (csPCa) and investigate its clinical efficacy to reduce unnecessary prostate biopsies.

**Methods:**

A total of 847 patients from institute 1 were included in cohort 1 for model development. Cohort 2 included a total of 208 patients from institute 2 for external validation of the model. The data obtained were used for retrospective analysis. The results of magnetic resonance imaging were obtained using Prostate Imaging Reporting and Data System version 2.1 (PI-RADS v2.1). Univariate and multivariate analyses were performed to determine significant predictors of csPCa. The diagnostic performances were compared using the receiver operating characteristic (ROC) curve and decision curve analyses.

**Results:**

Age, prostate-specific antigen density (PSAD), and PI-RADS v2.1 scores were used as predictors of the model. In the development cohort, the areas under the ROC curve (AUC) for csPCa about age, PSAD, PI-RADS v2.1 scores, and the model were 0.675, 0.823, 0.875, and 0.938, respectively. In the external validation cohort, the AUC values predicted by the four were 0.619, 0.811, 0.863, and 0.914, respectively. Decision curve analysis revealed that the clear net benefit of the model was higher than PI-RADS v2.1 scores and PSAD. The model significantly reduced unnecessary prostate biopsies within the risk threshold of > 10%.

**Conclusions:**

In both internal and external validation, the model constructed by combining age, PSAD, and PI-RADS v2.1 scores exhibited excellent clinical efficacy and can be utilized to reduce unnecessary prostate biopsies.

**Supplementary Information:**

The online version contains supplementary material available at 10.1186/s12957-023-02959-1.

## Introduction

Prostate cancer (PCa) affects millions of males every year. It is one of the most common solid malignancies, and the prognosis varies greatly by age, race, genetic background, and stage of progression [1]. Prostate biopsy remains the gold standard for PCa diagnosis. However, the imprecise localization of cancer focus by systematic biopsy has caused the overdiagnosis of clinically unimportant diseases and the underdiagnosis of clinically significant cancer [2]. Although invasive multiparametric magnetic resonance imaging-ultrasound (mpMRI-US) fusion biopsy has been demonstrated to improve the detection of clinically significant PCa (csPCa) and is more frequently used in clinical practice [3], the biopsy is associated with numerous complications, including bleeding, infection, and lower urinary tract symptoms [4]. Therefore, the reduction of prostate biopsy is crucial in males with low-grade or no PCa.

mpMRI has high sensitivity and specificity in detecting PCa and is essential for risk stratification of naïve patients, active surveillance for low-risk patients, and monitoring recurrence following definitive therapy [5]. The current standardized scheme for interpreting mpMRI is Prostate Imaging Reporting and Data System version 2.1 (PI-RADS v2.1) [6]. The major challenges in the clinical application of this approach are the low positive predictive value (PPV) and moderate interreader and intercenter reproducibility [7]. Although the prostate-specific antigen (PSA) has been used extensively as a PCa screening tool, its usage as a serum marker has drawbacks, such as an inability to accurately distinguish between benign and malignant conditions [8]. PSA density (PSAD) has long had the potential to improve the diagnostic utility of serum PSA alone by improving specificity while maintaining sensitivity; however, it has not been widely adopted in clinical practice. The construction of new multivariate risk prediction tools, which include the mpMRI suspicion score and PSAD, was largely based on the European or American populations. The European Randomised Study of Screening for Prostate Cancer Risk Calculator (ERSPC-RC) and the Prostate Cancer Prevention Trial Risk Calculator (PCPT-RC) are the calculators that have been the most extensively validated and compared. The ERSPC-RC and the PCPT-RC have recently been tested in some Asian cohorts; however, their performance is poor, particularly when compared to models developed in those countries [9]. These two risk calculators may not apply to Asian populations owing to differences in genetics, environment, imaging methods, and observers [10].

This study aimed to combine MRI and clinical data to construct a predictive model for csPCa and to investigate its clinical utility in avoiding unnecessary biopsies.

## Materials and methods

### Study population

This was a retrospective observational study approved by the local institutional review board. Initially, 1203 patients who underwent systematic transrectal ultrasound (TRUS)-guided and MRI-targeted prostate biopsies in institution 1 (First Hospital of Shanxi Medical University, Taiyuan, China) between January 2018 and June 2021 were included for model development. All patients underwent evaluation for elevated serum PSA levels (4 ng/mL) or abnormal results of a digital rectal examination (DRE) or TRUS. Exclusion criteria were previous prostate biopsy, prior treatment for PCa (prostatectomy, radiotherapy, focal therapy, hormonal therapy, etc.), and incomplete data. Finally, 847 patients were enrolled for evaluation (Supplementary Fig. 1).

A total of 317 patients in the external validation cohort underwent biopsies between May 2019 and January 2022 from institution 2 (Shanxi Bethune Hospital, Taiyuan, China). The biopsy indications and exclusion criteria were the same as those for the development queue. Of the 317 patients, 208 were enrolled in cohort 2 (Supplementary Fig. 2).

### MRI and PSAD

All MRI examinations of patients of the two institutions were performed using a 3-T scanner equipped with a pelvic 16- or 32-channel phased-array coil. The approach included T2-weighted image (T2WI), diffusion-weighted imaging (DWI), and dynamic contrast-enhanced (DCE). The DWI sequences were obtained with *b*-values of 0 and 1500. These images were stored in a picture archiving and communication system and analyzed by one radiologist of each institution (L. W. for institution 1 and L. Q. for institution 2). They were all blinded to the clinical details and had more than 15 years of prostate MRI experience.

Based on PI-RADS v2.1, a 3-point scale was used to assess the MRI diagnostic: grade 1 (PI-RADS 1–2 scores): low probability; grade 2 (PI-RADS 3 scores): equivocal; and grade 3 (PI-RADS 4–5 scores): high likelihood. T2WI was used to calculate prostate volume (PV) as follows: π/6 × length × height × width. PSAD was defined as serum total PSA divided by MRI-PV and was divided into four grades with a cut-off point of 0.15 ng/mL/mL.

### Biopsy procedure

All males from the two institutions underwent 12-core systematic biopsies. After obtaining the systematic biopsies, all patients from institution 1 underwent targeted biopsies, utilizing at least two biopsy cores per suspicious lesion identified on an MRI. Per the International Society of Urological Pathology standards, biopsy specimens and Gleason scores were evaluated by a pathologist and reviewed by another senior pathologist. csPCa was defined as a Gleason score ≥ 3 + 4.

### Statistical analysis

Quantitative variables were described using mean ± standard deviation (SD) or median (interquartile range), whereas qualitative variables were described as percentages (%). The correlations of variables were evaluated with Spearman’s rank correlation. For comparison of continuous variables, the Welch *t*-test or the Mann-Whitney-Wilcoxon test was used as a nonparametric alternative. A chi-square or Fisher exact test was applied to compare proportions. Binary logistic regression was used in both univariate and multivariate analyses to determine significant csPCa predictors. Bootstrap resampling (1000 samples) was used for internal validation of the model. The diagnostic performances of the predictive model and individual variables were assessed using receiver operating characteristic (ROC) curve analysis and compared using the areas under the ROC curves (AUC). The Youden index was used to determine the cut-off threshold. The net benefits of the model and individual variables were analyzed using decision curve analysis (DCA) [11]. *P* < 0.05 was considered statistically significant. All analyses were performed with SPSS software (version 22.0. IBM) and R version 4.1.2.

## Results

### Study populations

The development cohort (cohort 1) consisted of 847 patients from institution 1. Of the include patients, 367 (43.32%) had PCa, of which 305 (36.01%) had csPCa. In the external validation cohort (cohort 2), 104 (50%) of the 208 patients presented with PCa, of which 98 (47.1%) had csPCa. Patient demographics of the two cohorts were summarized in Table 1. The two cohorts had similar mean age, median PSA and its derivatives, and biopsy results; however, cohort 2 had a lower proportion of PI-RADS grade 2 (PI-RADS 3 scores).

**Table 1:** Patient demographics of development and validation cohorts

Variable	Cohort 1				Cohort 2				***P***
	All	No csPCa	Yes csPCa	***P***_**1**_	All	No csPCa	Yes csPCa	***P***_**2**_	
Case, *n* (%)	847	542 (63.99)	305 (36.01)		208	110 (52.9)	98 (47.1)		
Age, years (mean ± SD)	69.29 ± 8.45	67.51 ± 8.1	72.44 ± 8.1	< .001	70.28 ± 7.96	68.73 ± 7.61	72.02 ± 8.02	0.003	0.113
TPSA, ng/ml, M (IQR)	14.6 (8.6–32.0)	11.8 (7.7–18.2)	39.6 (14.7–104.3)	< .001	14.2 (9.3–35.7)	11.4 (7.6–20.2)	27.9 (12.6–79.3)	< .001	0.410
F/T, %, M (IQR)	13.8 (8.8–20.0)	14.5 (8.4–20.5)	12.6 (7.8–18.4)	0.064	14.3 (9.8–19.6)	15.6 (12.1–21.0)	11.7 (8.6–17.5)	0.002	0.224
PV, ml, M (IQR)	54.1 (38.5–82.4)	60.7 (43.5–89.9)	45.1 (30.9–63.9)	< .001	52.5 (36.9–79.1)	73.5 (49.1–94.6)	44.1 (32.9–62.4)	< .001	0.661
PSAD, ng/ml/ml, M (IQR)	0.26 (0.14–0.71)	0.19 (0.10–0.30)	0.88 (0.40–2.30)	< .001	0.27 (0.15–0.82)	0.16 (0.11–0.25)	0.68 (0.33–2.06)	< .001	0.342
PSAD < 0.15, *n* (%)	219 (25.86)	200 (36.90)	19 (6.23)		50 (24.04)	45 (40.91)	5 (5.10)		
0.30 > PSAD ≥ 0.15, *n* (%)	256 (30.22)	214 (39.48)	42 (13.77)		56 (26.92)	39 (35.45)	17 (17.35)		
0.45 > PSAD ≥ 0.30, *n* (%)	96 (11.33)	58 (10.70)	38 (12.46)		19 (9.13)	8 (7.27)	11 (11.22)		
PSAD ≥ 0.45, *n* (%)	276 (32.59)	70 (12.92)	206 (67.54)		83 (39.90)	18 (16.36)	65 (66.33)		
PI-RADS v2.1 grade, *n* (%)				< .001				< .001	< .001
1–2	247 (29.16)	240 (44.28)	7 (2.30)		71 (34.13)	64 (58.18)	7 (7.14)		
3	241 (28.45)	212 (39.11)	29 (9.51)		39 (18.75)	30 (27.27)	9 (9.18)		
4–5	359 (42.38)	90 (16.61)	269 (88.20)		98 (47.12)	16 (14.55)	82 (83.67)		
Biopsy results, *n* (%)				< .001				< .001	0.093
No cancer	481 (56.79)				104 (50.00)				
Gleason score < 7	61 (7.20)				6 (2.88)				
Gleason score 7	89 (10.51)				29 (13.94)				
Gleason score 8	83 (9.80)				23 (11.06)				
Gleason score 9	92 (10.86)				31 (14.90)				
Gleason score 10	41 (4.84)				15 (7.21)				

*TPSA* Total PSA, *F/T* Proportion of free PSA, *PV* Prostate volume, *PSAD* PSA density

### Prediction model development

**Fig. 1:**
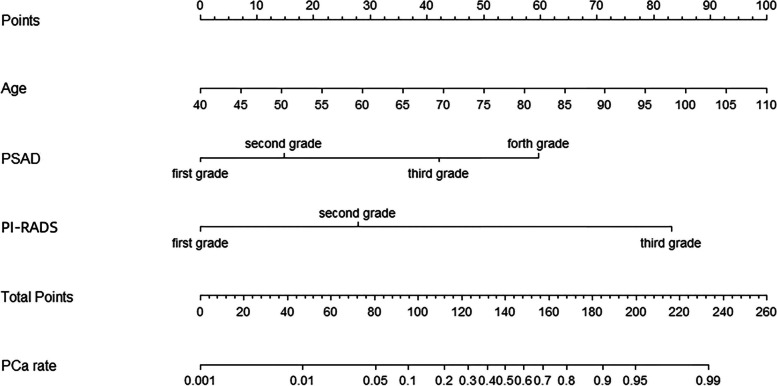
Risk model to predict csPCa including age, PSAD, and PI-RADS v2.1 scores. The regression equation was as follows: *Logit (p) = −13.839 + 0.069 × age + 1.026 × PSAD + 2.235 × PI-RADS grade*

**Fig. 2:**
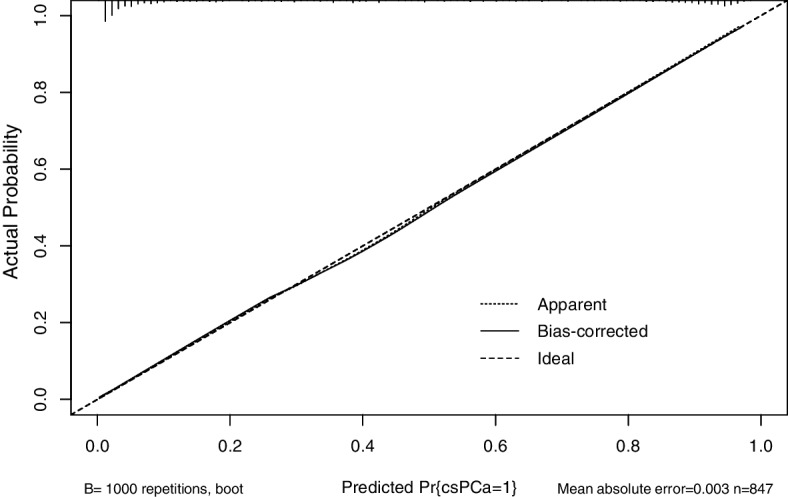
Calibration plot of the model

In univariate analysis, variables such as older age (67.51 vs 72.44, *P* < 0.001), elevated PSA (11.8 vs 39.6, *P* < 0.001), smaller PV (60.7 vs 45.1, *P* < 0.001), higher PSAD (0.19 vs 0.88, *P* < 0.001), and higher PI-RADS grade (16.61% vs 88.20%, *P* < 0.001) were associated with csPCa. Based on the multivariable binary logistic analysis, the model was eventually constructed by age, PSAD, and PI-RADS v2.1 score. Only the PSAD with the best prediction performance was included in the model owing to the correlation between total PSA, free PSA/total PSA, PV, and PSAD. A nomogram predicting the absence of csPCa was developed based on the three parameters (Fig. 1). The C-index of the model was 0.9379. The model for predicting the presence of csPCa exhibited excellent calibration (Fig. 2).

**Fig. 3:**
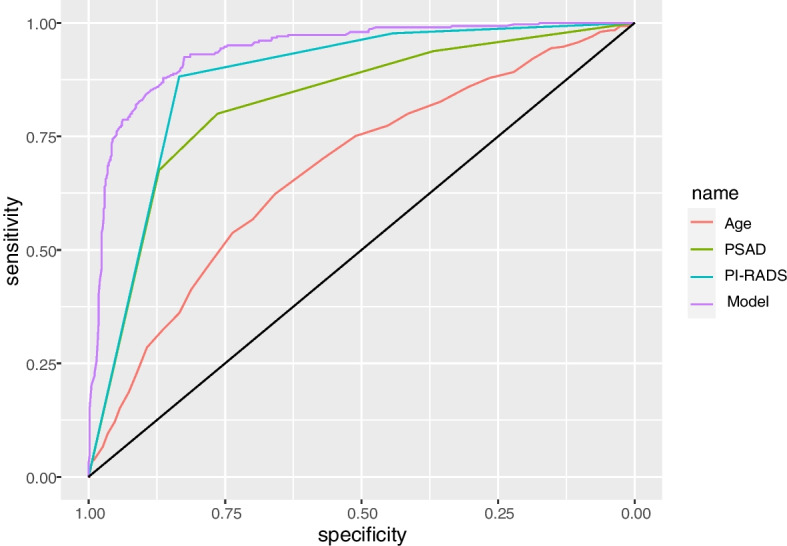
ROC curves of age, PSAD, PI-RADS grade, and the model for csPCa in cohort 1. Their AUC values were 0.675, 0.823, 0.875, and 0.938, respectively

ROC analysis revealed that the model had a larger AUC in comparison with other parameters. The AUC values of age, PSAD, PI-RADS grade, and the model were 0.675 (95% confidence interval (CI), 0.637–0.712), 0.823 (95% *CI*, 0.793–0.853), 0.875 (95% *CI*, 0.850–0.899), and 0.938 (95% *CI*, 0.922–0.955), respectively (Fig. 3). The predictive accuracy of the model was significantly higher than that of the others. The optimal cut-off value of the model was 0.3, and its sensitivity and specificity values were 0.925 and 0.825, respectively.

**Fig. 4:**
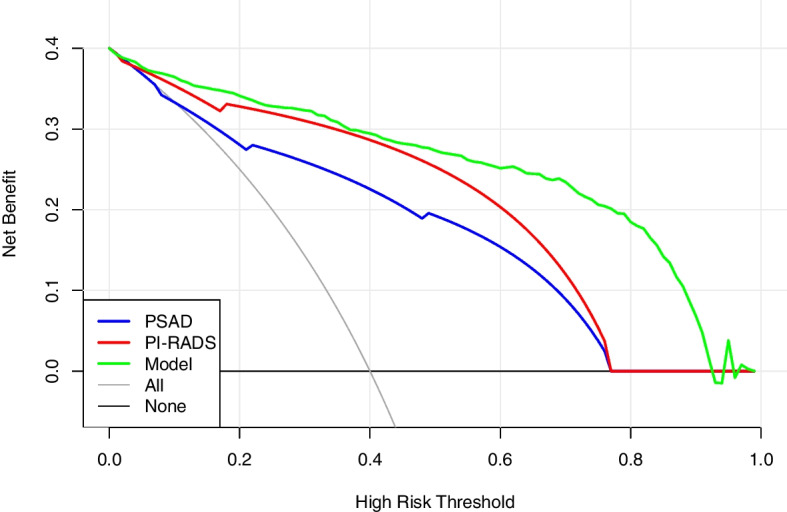
Decision curves for csPCa of PSAD, PI-RADS grade, and the model in cohort 1

DCA demonstrated that the model had higher net benefits than PI-RADS grade and PSAD in a wide range of probability thresholds, indicating that the clinical value of the model was superior to that of PI-RADS grade or PSAD alone (Fig. 4).

### External validation

In cohort 2, the predictive model evaluated that 86 patients with a prediction probability of ≥ 0.3 had csPCa, and 94 patients with a prediction rate of < 0.3 did not have csPCa. Furthermore, 16 patients were false positive, and 12 were false negative. If the prediction probability was close to one, the patient was more susceptible to having csPCa, whereas if that was close to zero, the patient was susceptible to being free of csPCa. The prediction results revealed that the sensitivity, specificity, PPV, negative predictive value, and accuracy of the model were 87.76%, 85.45%, 84.31%, 88.68%, and 86.54%, respectively (Table 2).

**Table 2:** Sensitivity, specificity, PPV, NPV, and AUC of the model in the two cohorts

	Sensitivity, %	Specificity, %	PPV, %	NPV, %	AUC (95% ***CI***)
Cohort 1	92.50	82.50	74.60	95.10	0.936 (0.922–0.955)
Cohort 2	87.76	85.45	84.31	88.68	0.914 (0.914–0.955)

**Fig. 5:**
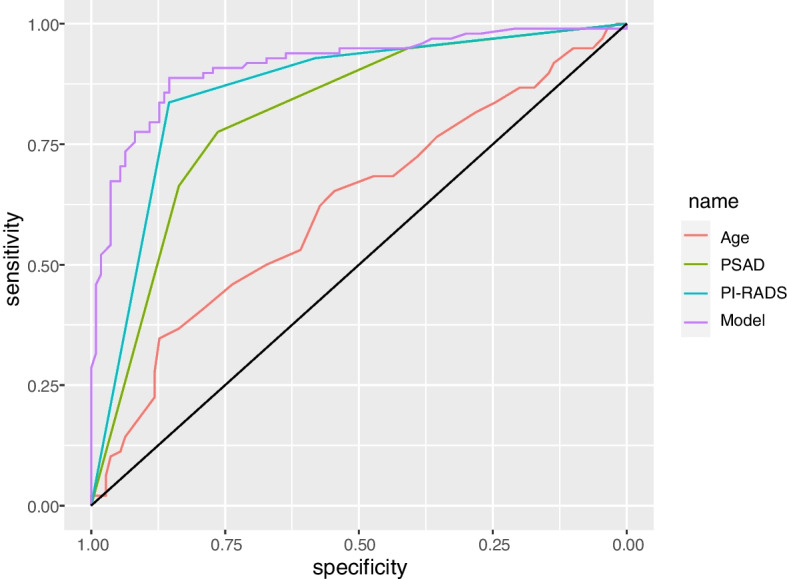
ROC curves of age, PSAD, PI-RADS grade, and the model for csPCa in cohort 2. Their AUC values were 0.619, 0.811, 0.863, and 0.914, respectively

ROC analyses revealed that the AUC values of age, PSAD, PI-RADS grade, and the model were 0.619 (95% *CI*, 0.542–0.696), 0.811 (95% *CI*, 0.751–0.871), 0.863 (95% *CI*, 0.809–0.916), and 0.914 (95% *CI*, 0.873–0.955), respectively (Fig. 5). The results indicated that the diagnostic accuracy of the model remained higher than that of the other parameters in the external validation cohort.

**Fig. 6:**
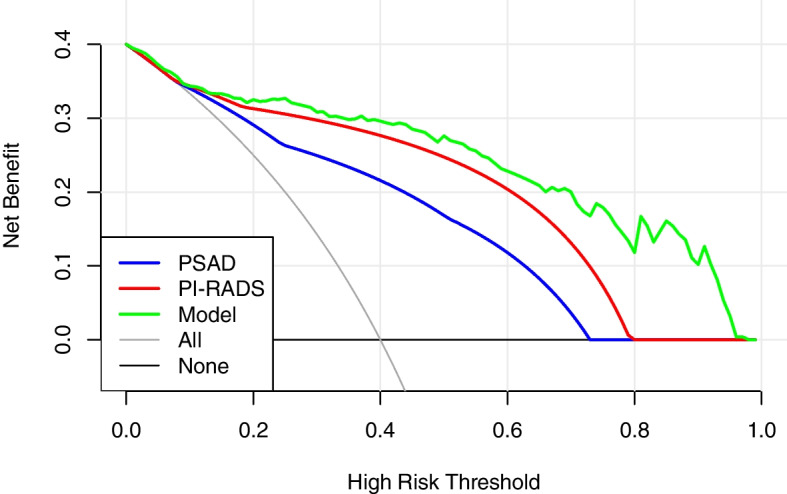
Decision curves for csPCa of PSAD, PI-RADS grade, and the model in cohort 2

DCA suggested that the model still had better clinical utility than PI-RADS grade and PSAD in the range above the 10% risk threshold (Fig. 6). The net benefit was calculated with the formula = $\frac{TP}{N}-\frac{FP}{N}\times \frac{P_t}{1-{P}_t}$, where *TP* was the number of true-positive decisions, *FP* was the number of false-positive decisions, *N* was the total number of patients, and *P*_t_ was the threshold probability. For example, at a 20% risk threshold, the net benefits of PSAD, the PI-RADS score, and the model were 0.369, 0.382, and 0.394, respectively. Overall, the model did not reduce the number of biopsies within the 10% risk threshold. However, with no increase in the number of patients with csPCa who missed the biopsy, at the risk threshold of 20%, 30%, and 40%, the model reduced the number of unnecessary biopsies by 22.12%, 31.73%, and 36.54%, respectively (Table 3).

**Table 3:** The net benefit and the proportion of unnecessary biopsies reduced for PSAD, mpMRI, and the model in cohort 2

Risk threshold, %	Net benefit				Avoided biopsies		
	All biopsies	PSAD	MRI	Model	PSAD	MRI	Model
10	0.412	0.412	0.413	0.405	0	0.45%	−6.75%
20	0.339	0.369	0.382	0.394	12.04%	17.32%	22.12%
30	0.245	0.313	0.342	0.381	16.03%	22.91%	31.73%
40	0.119	0.239	0.290	0.362	18.03%	25.37%	36.54%

## Discussion

Prostate biopsies are necessary to confirm the diagnosis of PCa in patients who experience an increase in the total PSA. Consequently, numerous patients with rising PSA undergo unnecessary biopsies. Currently, European Association of Urology (EAU) guidelines recommend prostate biopsy for patients with a PI-RADS score ≥ 3. A compelling example is the PROMIS study of prostate MRI, which demonstrated an mpMRI sensitivity of approximately 93% in detecting csPCa [2]. However, a recent multicenter study revealed that there is still a significant degree of variation: the PPV of PI-RADS score of ≥ 3 for detecting csPCa ranged from 27 to 48% in 26 centers [12]. In this study, the PI-RADS v2.1 score had a PPV of 49.67% and a sensitivity of 97.70% in cohort 1 and a PPV of 66.42% and a sensitivity of 92.86% in cohort 2. Although MRI exhibited high sensitivity, its PPV was low, which resulted in a significant proportion (35.66% in cohort 1 and 22.12% in cohort 2) of unnecessary biopsies. Additionally, since PI-RADS score was an important predictor of the model and its PPVs differed between the two cohorts, the PPV of the model might be overestimated in cohort 2. A study reported that patients with a PI-RADS score of 1 or 2 were significantly younger, had lower PSA levels, and had fewer biopsy-positive cores than those with PI-RADS scores of 3–5 [13]. In this study, similar characteristics were observed by analyzing each subgroup of MRI scores in the two cohorts. In comparison with the strategy of using PI-RADS score 3 as the biopsy standard, by combining PI-RADS score with age and PSAD, the current predictive model reduced the number of unnecessary biopsies by 14% in cohort 1 and 12% in cohort 2, at the cost of only 1.89% and 2.40% more missing patients in cohorts 1 and 2, respectively.

Several studies have reported that PSAD and prostate MRI suspicion scores were significant independent predictors of csPCa at biopsy [14]. Numerous studies have suggested using a PSAD cut-off of 0.15 ng/mL/mL to identify patients with negative MRI who nonetheless require a prostate biopsy [15]. The most recent EAU guideline specifically made reference to this cutoff for the same reason. The PSAD test performance for detecting PCa has reported a specificity of 0.63–0.74 and a sensitivity of 0.70–0.79 at the threshold of 0.15 ng/mL/mL [13]. However, a recent study with 8974 prostate biopsy samples revealed that the use of a PSAD cutoff of 0.15 ng/mL/mL to recommend a prostate biopsy to patients with negative MRI is justified only in the case of very low MRI accuracy. Additionally, a higher cutoff of at least 0.20 should be employed for the average MRI [16]. In this study, ROC analysis revealed that the optimal cut-off value of PSAD in cohort 1 was 0.37 ng/mL/mL, and the sensitivity and specificity were 0.757 and 0.832, respectively, which were similar to those in cohort 2. This high cut-off value of PSAD may be caused by PSA levels above 4 ng/mL in 97% of the population under study. Additionally, some extreme values were not eliminated, and all PSAD values were graded into four levels. This study also demonstrated that high-grade PSAD was associated with a higher Gleason score. Previous studies have demonstrated a correlation between PSAD and higher pathological staging and PCa aggressiveness [17]. Another study reported that between initial prostate biopsy and prostatectomy, PSAD was the strongest predictor of tumor upgrading in the Gleason score [18]. Therefore, PSAD is not only an important predictor of biopsy outcome but may also help suggest clinically significant and aggressive PCa.

Owing to the predictive value of mpMRI and PSAD in PCa diagnosis, some new multivariate risk prediction tools have recently been constructed. To improve their predictive value, some used extra input variables such as age, PV, free PSA, family history, race, and prior negative biopsy [19–22]. These MRI risk prediction models were examined and contrasted using a systematic review. They discovered that all of their MRI models used PSA and DRE as individual predictive input variables in, and these MRI models exhibited high accuracy in comparison with baseline models, with AUC values ranging from 0.78 to 0.93 [23]. A near-term review reported that PCa risk increases strongly with age in over 85% of newly diagnosed individuals who are > 60 years. In this study population of 1055 in both cohorts, 91% of all patients with PCa were older than 60 years [1]. The current model introduced age as a predictor, and the AUC value of this model was as high as 0.914 in external validation. Additionally, Schoots and coworkers compared the net benefits of previous MRI models, which ranged from 0.100 to 0.347 at a 20% risk threshold. DRE and TRUS, as commonly reported risk factors, have poor sensitivity and high inter-observer variability for csPCa [24] and were thus not included in the model. Higher than any of them was the model established in this study, with a net benefit of 0.394 at a 20% risk threshold.

Additionally, this study calculated the net benefit and the proportion of unnecessary biopsies reduced for PSAD, mpMRI, and the model. Positive PSAD was defined as ≥ 0.15 ng/mL/mL, and positive MRI was defined as ≥ 3 points. The results revealed that, in the range above the 10% risk threshold, the model demonstrated higher clinical efficacy and a significant reduction in unnecessary biopsies.

This study had several limitations. First, owing to the small population recruited from institution 2, it may not be sufficient to accurately depict the clinical diagnostic performance of the model. Second, the model requires more extensive external validation by multiple agencies. Third, most of the population included in this study has a PSA level > 4 ng/mL. Owing to the selection bias, the applicability of this model in patients with low PSA values may be limited. Fourth, since the MRI from each institution was evaluated by a single radiologist, the inter-observer reliability could not be assessed. Last, all groups used biopsy results to determine whether they had csPCa. Owing to the limitations of biopsy technology, some patients might have been missed.

## Conclusions

This study constructed a csPCa prediction model using age, PSAD, and PI-RADS v2.1 score based on mpMRI, which had good clinical utility and performed well in independent external validation. The model can be used to reduce the number of unnecessary prostate biopsies in Asian patients with significantly elevated PSA levels while accurately identifying most of the patients with csPCa.

## Supplementary Information

**Additional file 1: Supplementary Fig. 1.** Inclusion and exclusion criteria for cohort 1.

**Additional file 2: Supplementary Fig. 2**. Inclusion and exclusion criteria for cohort 2.

## Data Availability

The datasets used and/or analyzed during the current study are available from the corresponding author on reasonable request.
