# Supplementary figures and images for: Developing a predictive model for clinically significant prostate cancer by combining age, PSA density, and mpMRI
Source: World J Surg Oncol. 2023 Mar 7;21:83. doi: 10.1186/s12957-023-02959-1 (PMC9990202; doi:10.1186/s12957-023-02959-1)

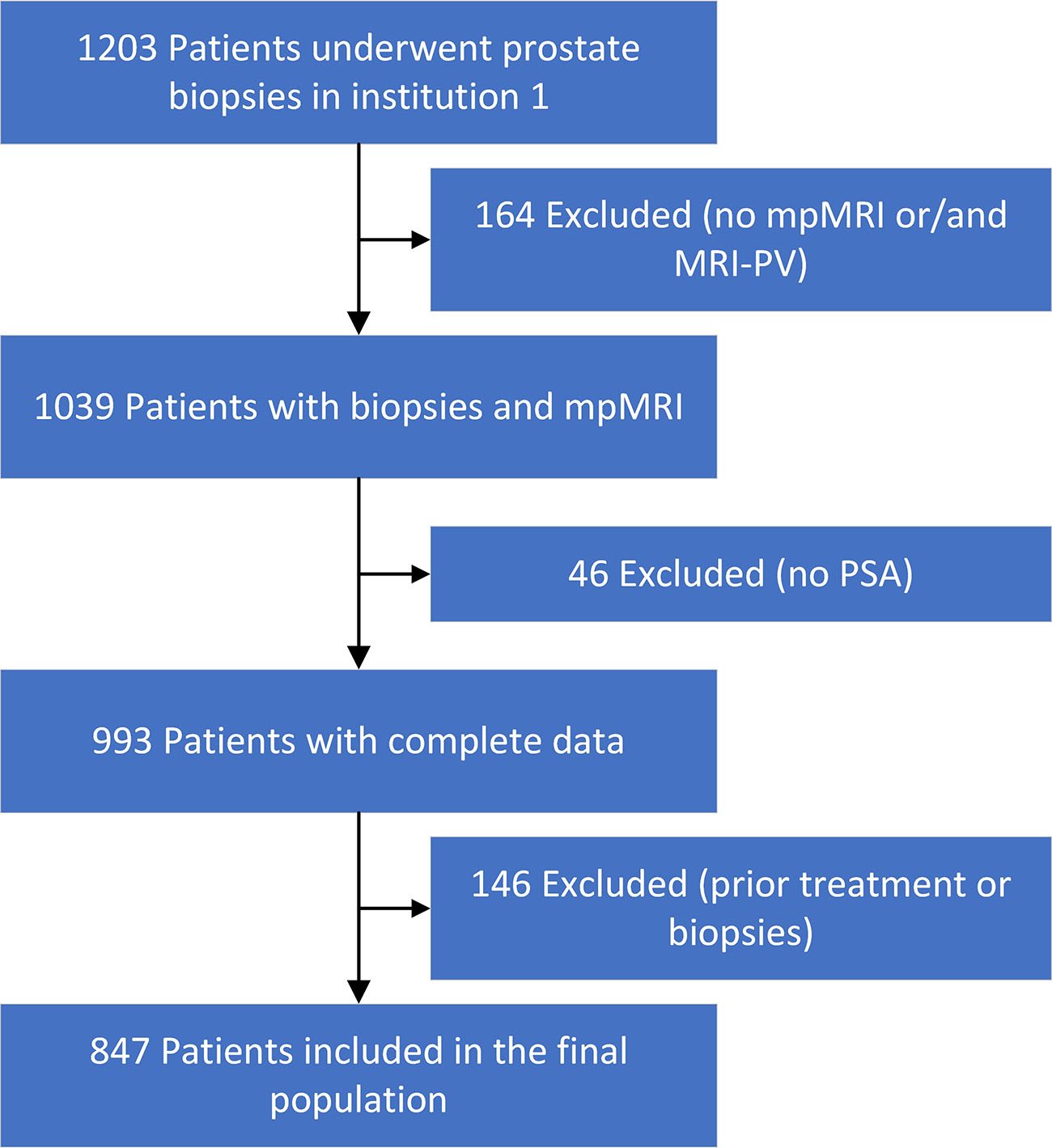

Supplement: Supplementary file 1 — Additional file 1: Supplementary Fig. 1. Inclusion and exclusion criteria for cohort 1. [file 12957_2023_2959_MOESM1_ESM.jpg]

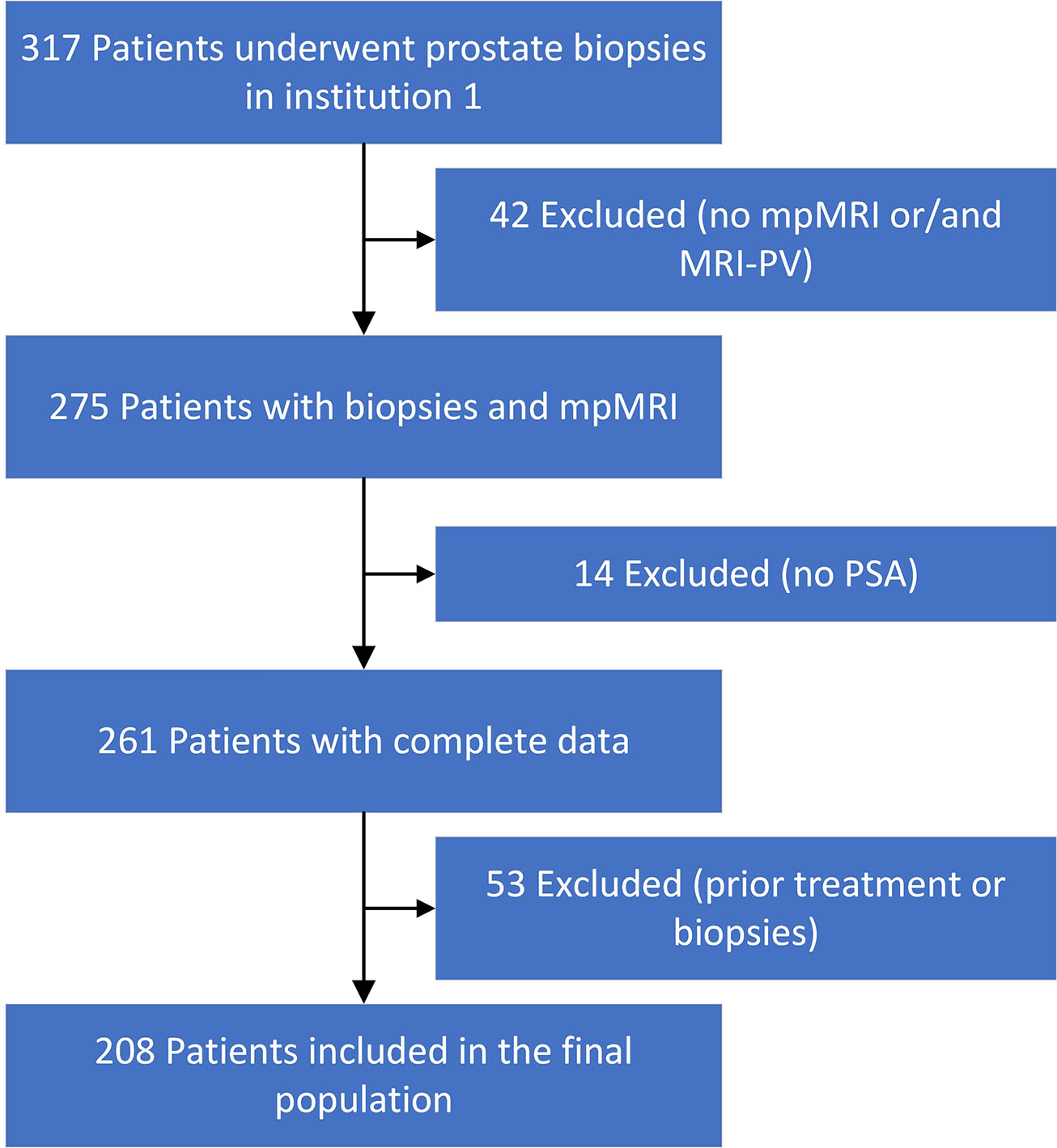

Supplement: Supplementary file 2 — Additional file 2: Supplementary Fig. 2. Inclusion and exclusion criteria for cohort 2. [file 12957_2023_2959_MOESM2_ESM.jpg]
